# Addressing the Need for Universal Cognitive Assessment Among Older Adults With Congestive Heart Failure

**DOI:** 10.7759/cureus.62838

**Published:** 2024-06-21

**Authors:** Myrna K Serna, Shilpa Rajagopal, Sunil K Sahai, Mukaila Raji

**Affiliations:** 1 Department of Internal Medicine-Division of General Medicine, University of Texas Medical Branch at Galveston, Galveston, USA; 2 Department of Internal Medicine, University of Texas Medical Branch at Galveston, Galveston, USA; 3 Department of Internal Medicine, Division of Geriatrics and Palliative Medicine, University of Texas Medical Branch at Galveston, Galveston, USA

**Keywords:** mortality, geriatrics, heart failure, dementia, cognitive impairment

## Abstract

Congestive heart failure (CHF) and cognitive impairment (CI) are common in older age and often occur together. However, CI is often unrecognized leading to more hospitalizations and increased morbidity and mortality in patients with co-occurring CHF and CI. Universal screening can help identify these patients earlier and the use of the Institute of Healthcare Improvement’s (IHI) 4Ms Framework (i.e. “What Matters, Medication, Mentation, and Mobility”) can serve as a tool for providers to meet patients’ needs surrounding goals of care, medication regimens, mental and emotional well-being, and mobility capabilities through a social determinants of health lens. Providers should engage in serious illness conversations early to honor patient preferences, reduce polypharmacy, use a validated instrument to assess cognition such as the Mini-Cog or Functional Activities Questionnaire, and assess the need for assistance completing activities of daily living (ADLs) and instrumental activities of daily living (IADLs). Consultation with social work is highly recommended given the complexity of the medical and social needs of newly discharged cognitively impaired CHF patients and the need to optimize the use of all available community resources.

## Editorial

Congestive heart failure (CHF) and cognitive impairment (CI) are common in older age and often occur together. CHF has a prevalence of more than 10% in patients aged 70 years or older [1] and is the most common cause of hospitalization in this population. Thirty-two percent of patients with CHF have mild CI, but rates have been found to be as high as 73% [1]. Amid a growing elderly population, the number of patients with dementia is anticipated to double every 20 years worldwide [1]. Considering the high rate of co-occurrence of CI and CHF, the goal of this article is to highlight the need to assess cognitive status in older patients with CHF and discuss how such assessment can guide shared clinical decision-making surrounding goals of CHF care using social determinants of health (SDoH) framework.

Pathogenesis of CI in patients with CHF

The pathogenesis of CI in relation to CHF is multifactorial and includes increased inflammatory changes, heightened risk of strokes and transient ischemic attacks, and lower levels of cerebral blood flow due to overall decreases in cardiac output, all of which may predispose individuals to CI. Additional factors such as polypharmacy and decreased social and physical activity can also contribute to observable declines in cognitive functioning among individuals with chronic, lifestyle-limiting disease states like CHF.

Unrecognized CI is burdensome

Unrecognized CI impedes and worsens CHF care, while sub-optimally managed CHF exacerbates cognitive function. Patients with both conditions have more hospitalizations and increased morbidity and mortality compared to patients with CHF who are cognitively intact. In a study to determine whether 30-day CHF readmissions were associated with cognitive impairment, 68% of 70-year-olds with a primary diagnosis of HF were found to have cognitive impairment, with fewer than 10% having had prior documentation of CI in their medical record [2]. Moreover, management of CHF is complex and includes multiple drug therapies, changes in diet and lifestyle, and close monitoring of weight, blood pressure, and symptoms requiring frequent follow-up appointments. This complexity can make it difficult for any patient to follow the management plan but is especially burdensome for those with CI. In addition, many CHF patients have additional chronic conditions, adding further intricacies to their management plans. It is therefore of utmost importance to consider assessing CI in patients with CHF, especially among those who have faced challenges with following the medical plan and who are already experiencing poor outcomes, including multiple hospitalizations. Findings from such assessments can guide medication regimen choice (e.g., use of a one-a-day “polypill” to reduce pill burden and improve adherence), inform sites of post-acute care discharge (e.g., skilled nursing facility where medications are administered by staff), and influence the use of technology for remote heart monitoring of cognitively impaired CHF patients being discharged from the hospital.

Adapting the Institute for Healthcare Improvement 4Ms Framework to Optimize Care for Cognitively Impaired CHF Patients

**Figure 1:**
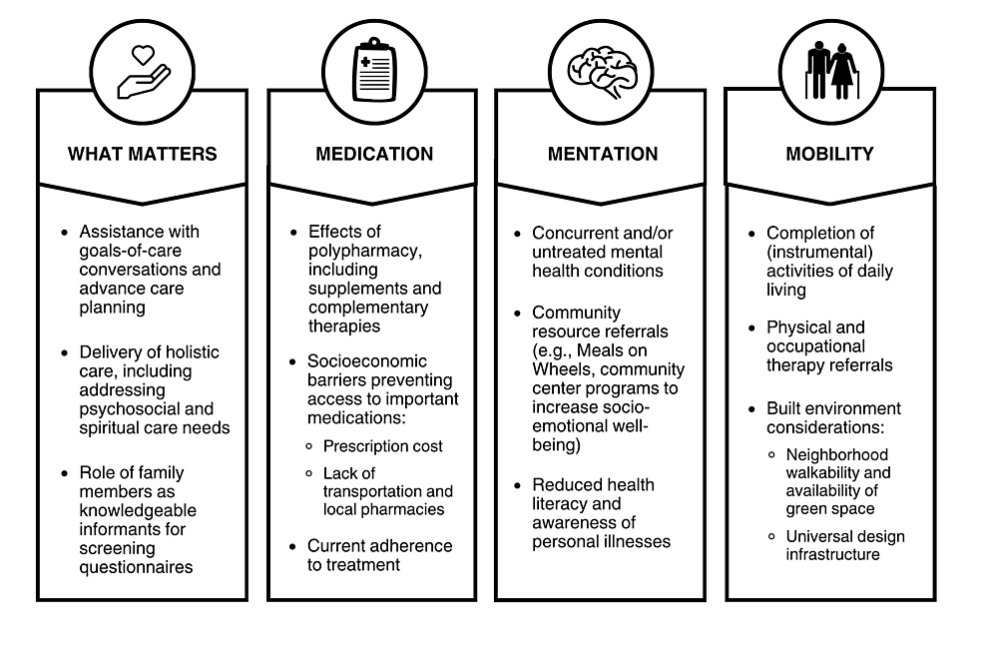
Priority considerations using the IHI 4Ms Framework for patients with co-occurring CHF and CI CHF: congestive heart failure; CI: cognitive impairment The image was created by the authors of this article. The icons at the top of the figure are from the free Canva graphics. The figure was included as part of a poster presented previously by the authors of this article at the 2023 UTMB Annual Forum on Aging (Rajagopal S, Serna MK, Pujari SH, Khan F, Kaushik V, Raji M. Addressing the Need for Universal Cognitive Testing Among Older Adults with Congestive Heart Failure. Poster presented at: 26th Annual Forum on Aging; 2023 October; Galveston, TX.)

To address the needs of older adults, the Institute for Healthcare Improvement (IHI) 4Ms Framework adopts the following criteria and care considerations: “What Matters, Medication, Mentation, and Mobility” [3]. Success in achieving the IHI Age-Friendly Health Systems goals requires a consideration of various SDoH: income, education, healthcare access and quality, the surrounding neighborhood and built environment, and overall social and community context. Adapting the 4M framework can serve as a meaningful approach to improving quality-of-life and outcomes in older adult patients with CHF and CI (Figure 1) [3].

What Matters

Providers should engage in advance care planning or serious illness conversations with patients who have chronic, life-limiting illnesses, including those with CHF and CI, to understand what matters most to these patients and make changes to the management plan that are aligned with patient wishes. CHF and CI are common co-occurring conditions with complex management, requiring intense social support and medical interventions. It is imperative that patient priorities are forefront as they progress in their illness. Advance care planning should also be considered for patients of all backgrounds, including Hispanic and Black patients who have reported having these important conversations less frequently.

If eliciting patient priorities is not possible due to more advanced dementia, speaking with the patient’s family or those closest to them to build prognostic awareness and discuss goals of care is important due to the high rate of burdensome interventions, including emergency department visits, hospitalizations, parenteral therapy, and high mortality rates, in this population. Palliative care and hospice should also be considered, as hospice is often underutilized among patients with heart failure, especially those with severe comorbidity and recurrent hospitalization and racial and ethnic minority populations. For hospitalized CHF patients with advanced dementia, early discussion with the patient’s proxy about long-term care placement and hospice is key to preventing recurrent hospitalizations and other burdensome end-of-life care interventions.

Medication

When managing patients with co-occurring CHF and CI, engaging and educating those closest to them upon discharge has been shown to substantially reduce readmissions. Home health services may also be considered in this patient population for medication management and blood pressure and weight monitoring. While difficult to do, polypharmacy and CNS-active medications should be avoided where possible, and the use of the “polypill” approach should be explored. Patients with economic instability, transportation constraints, and limited healthcare access may encounter difficulties with obtaining vital medications. Social work consults should be utilized to intentionally address these risk factors.

Mentation

To assess for presence of CI, a validated instrument to examine cognition can be used. Some tests can be completed within several minutes and are administered to either the patient (e.g., Mini-Cog) or to an informant (e.g., Functional Activities Questionnaire) [4]. Lengthier follow-up CI screening tests can be conducted based on need and the results from the Mini-Cog evaluation. Delirium assessment is especially important in hospitalized CHF patients, as clinical and laboratory findings from such assessments can reveal potentially treatable contributors of the acute cognitive change. The Confusion Assessment Method (CAM) is an example of a commonly used tool for delirium assessment [5].

Presence of co-occurring mental health disorders (e.g., depression, anxiety), substance use disorders (e.g., alcohol and opioid use disorder), sleep disturbances (e.g., sleep apnea), and other medical conditions with known mental health associations (e.g., hypothyroidism and vitamin B12 deficiency) should also be considered during CI evaluations [4]. Additionally, vision and hearing problems are very common in older age and should be routinely assessed.

Mobility

Mobility and activities of daily living (ADLs) are frequently suboptimal among cognitively impaired CHF patients. The need for assistance in both promoting mobility and completing ADLs and instrumental activities of daily living (IADLs) should be properly assessed. Providers should consider physical and occupational therapy options at home or at a short-stay, post-acute care facility such as a skilled nursing facility to optimize function and safety. This is not only important for CHF management but also serves to improve lifestyle interventions including exercise, cognitive stimulation, social engagement, and nutrition which are standard of care and first-line in patients with dementia [4].

Optimal use of all community resources is vital, including services such as Meals on Wheels and adult day care programs, to promote overall health and well-being. Consultations with social work and/or case management are highly recommended given the complexity of the medical and social needs of newly discharged cognitively impaired CHF patients - a population at high risk of CHF readmission.

Conclusion

The co-occurrence of CHF and CI highlights the need for evidence-based, holistic screening guidelines to identify instances of CI and accordingly help tailor treatment plans for this subset of older adult CHF patients. Universal screening of CI among older adults with CHF can help identify such patients earlier and improve standards of care. When conducting these assessments, the 4Ms Framework developed by the IHI can serve as a tool for providers to meet patient needs surrounding goals of care, medication regimens, mental and emotional well-being, and mobility capabilities through an SDoH lens.
